# The NIH Open Citation Collection: A public access, broad coverage resource

**DOI:** 10.1371/journal.pbio.3000385

**Published:** 2019-10-10

**Authors:** B. Ian Hutchins, Kirk L. Baker, Matthew T. Davis, Mario A. Diwersy, Ehsanul Haque, Robert M. Harriman, Travis A. Hoppe, Stephen A. Leicht, Payam Meyer, George M. Santangelo

**Affiliations:** 1 Office of Portfolio Analysis, Division of Program Coordination, Planning, and Strategic Initiatives, Office of the Director, National Institutes of Health, Bethesda, Maryland, United States of America; 2 UberResearch GmbH, Cologne, Germany

## Abstract

Citation data have remained hidden behind proprietary, restrictive licensing agreements, which raises barriers to entry for analysts wishing to use the data, increases the expense of performing large-scale analyses, and reduces the robustness and reproducibility of the conclusions. For the past several years, the National Institutes of Health (NIH) Office of Portfolio Analysis (OPA) has been aggregating and enhancing citation data that can be shared publicly. Here, we describe the NIH Open Citation Collection (NIH-OCC), a public access database for biomedical research that is made freely available to the community. This dataset, which has been carefully generated from unrestricted data sources such as MedLine, PubMed Central (PMC), and CrossRef, now underlies the citation statistics delivered in the NIH *iCite* analytic platform. We have also included data from a machine learning pipeline that identifies, extracts, resolves, and disambiguates references from full-text articles available on the internet. Open citation links are available to the public in a major update of *iCite* (https://icite.od.nih.gov).

## Abstract 2

In this Community Page article, authors from the National Institutes of Health describe the Open Citation Collection, a public domain citation database that covers articles indexed in PubMed. This free resource is made available through the *iCite* web interface at icite.od.nih.gov and through bulk downloads.

## Background

“If I have seen farther, it is by standing on the shoulders of giants,” wrote Issac Newton [1]. Science advances the frontier of knowledge by building on the discoveries described in the literature, and the provenance and spread of scientific discoveries is documented in the directed graph of the embedded citations [2,3]. Meta-research, frequently drawing on the historical citation record, seeks to apply scientific methods to further accelerate research by identifying potential improvements to research practices and organization [4,5]. Paywalled citation data remain locked behind restrictive licensing agreements, raising an unnecessary barrier to entry for investigators and blocking the increasingly common practice of data sharing in scientific articles that use this information. This state of affairs prevents many scientists from using comprehensive citation graphs in their research, reduces the robustness and reproducibility of the analyses that do use it, and hinders research in bibliometrics [6]. The Initiative for Open Citations [7,8] was a crucial step toward opening public access to this structured information, which has so far made public an estimated 55% of the reference links between documents indexed in CrossRef. Here, we describe a comprehensive, public domain citation graph for biomedical research made freely available to the community. This citation database is not a static snapshot but—as a part of our bibliometrics web service, *iCite* [9]—will be updated monthly.

While developing new science-of-science methodologies [10,11], the NIH Office of Portfolio Analysis (OPA) has carefully pursued unencumbered citation data resources that can be shared publicly. The NIH Open Citation Collection (NIH-OCC) described here underpins the *iCite* database that distributes citation metrics worldwide. Data sources include federally funded resources such as PubMed Central (PMC) [12], MedLine [13], and Entrez [14] from the National Library of Medicine (NLM); the community resource CrossRef [15]; and reference data harvested from full-text scientific papers that have been made freely available on the internet. These open access articles were identified either through explicit journal policies or through third-party aggregators such as Unpaywall [16]. Citation data can be visualized and downloaded through the *iCite* website [9]; the data can also be accessed via machine-readable Application Programming Interface (API) or bulk downloads.

In a call for open citation data from the scientometrics community [17,18], signatories noted the capacity of open data to improve transparency and reproducibility of analyses. Transparency is also an important goal for the NIH; link-level citation data have been disseminated through PMC, making transparent the flow of knowledge from earlier work to NIH-supported publications. With the release of this NIH-OCC for biomedicine at large, the subsequent work that draws upon NIH-supported discoveries is now visible as well, and barriers to entry for scientometric studies will be reduced. The science-of-science community has illustrated the high value of link-level citation data (as opposed to aggregated citation measures), e.g., using such information to discover principles of citation dynamics [19–21], quantify the influence of model organism research on human studies [22], and predict the transmission of knowledge from basic research into clinical studies [23]. Thus, comprehensive open citation data can both enable the attribution of scientific progress and convey foreknowledge that research discoveries will culminate in downstream applications.

## Description

*iCite* currently draws on PubMed for crucial article metadata, and this information is augmented with citation data from multiple sources. The NLM resolves citations from PMC to PubMed articles, disseminating these through a few resources (Entrez eLink, PMC full-text XML, and MedLine XML). We augment these citations with CrossRef citation data, which are processed by a citation resolver to identify additional PubMed-to-PubMed citations. For publications since 2010, the NIH-OCC has more citation links and is therefore more comprehensive than leading proprietary sources. Prior to 2010, a subset of historical articles (typically published during or before the early 2000s) have not been assigned DOIs and are therefore not captured in the CrossRef dataset. For this reason, we have further augmented these data sources with information from full-text articles that have been made freely available on the internet. We developed a prototype machine learning pipeline, described below, to identify, parse, and resolve references from such full-text articles for inclusion in the NIH-OCC. Finally, once citations are resolved, these are entered into our data processing pipelines for calculating downstream metrics like the Relative Citation Ratio [10] and the Approximate Potential to Translate [23].

**Fig 1:**
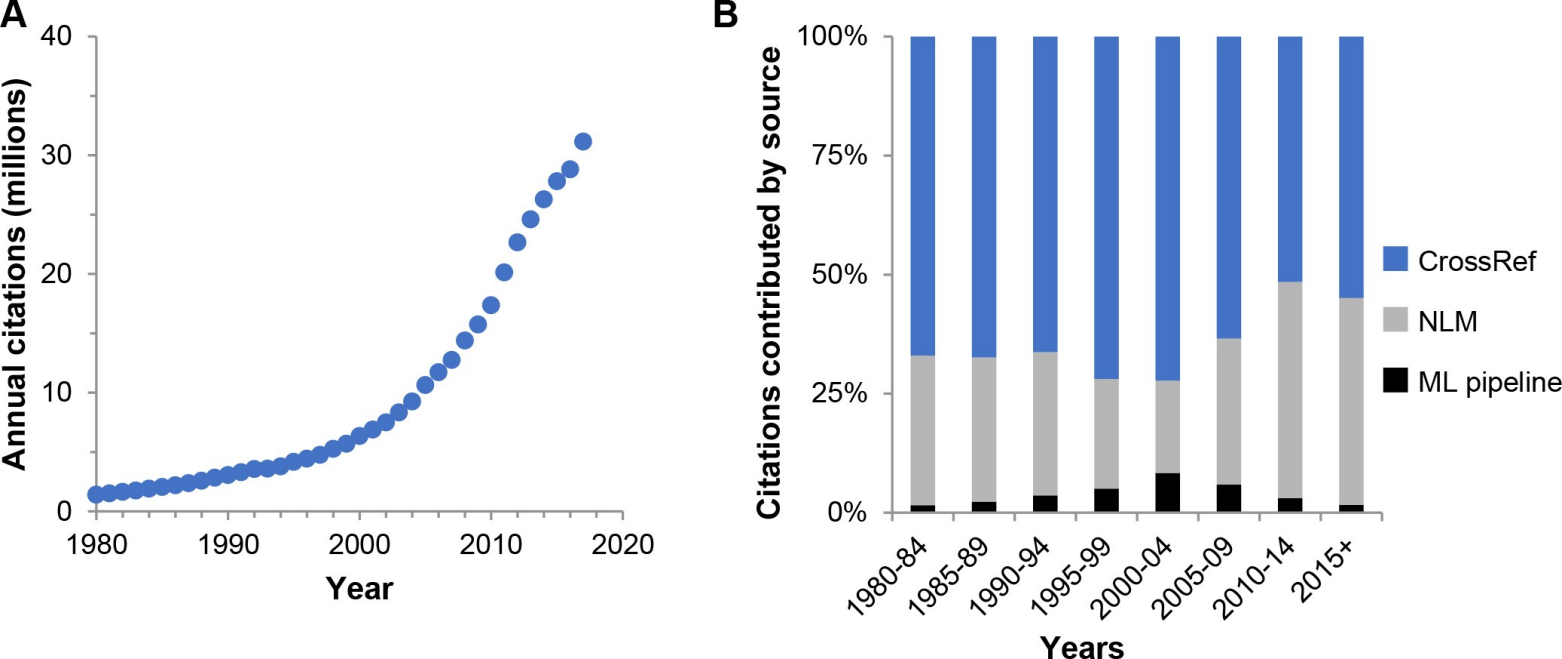
Citations in the NIH-OCC. (A) Citations per year. (B) Citation source by time period. ML, Machine Learning; NLM, National Library of Medicine; OCC, Open Citation Collection.

**Fig 2:**
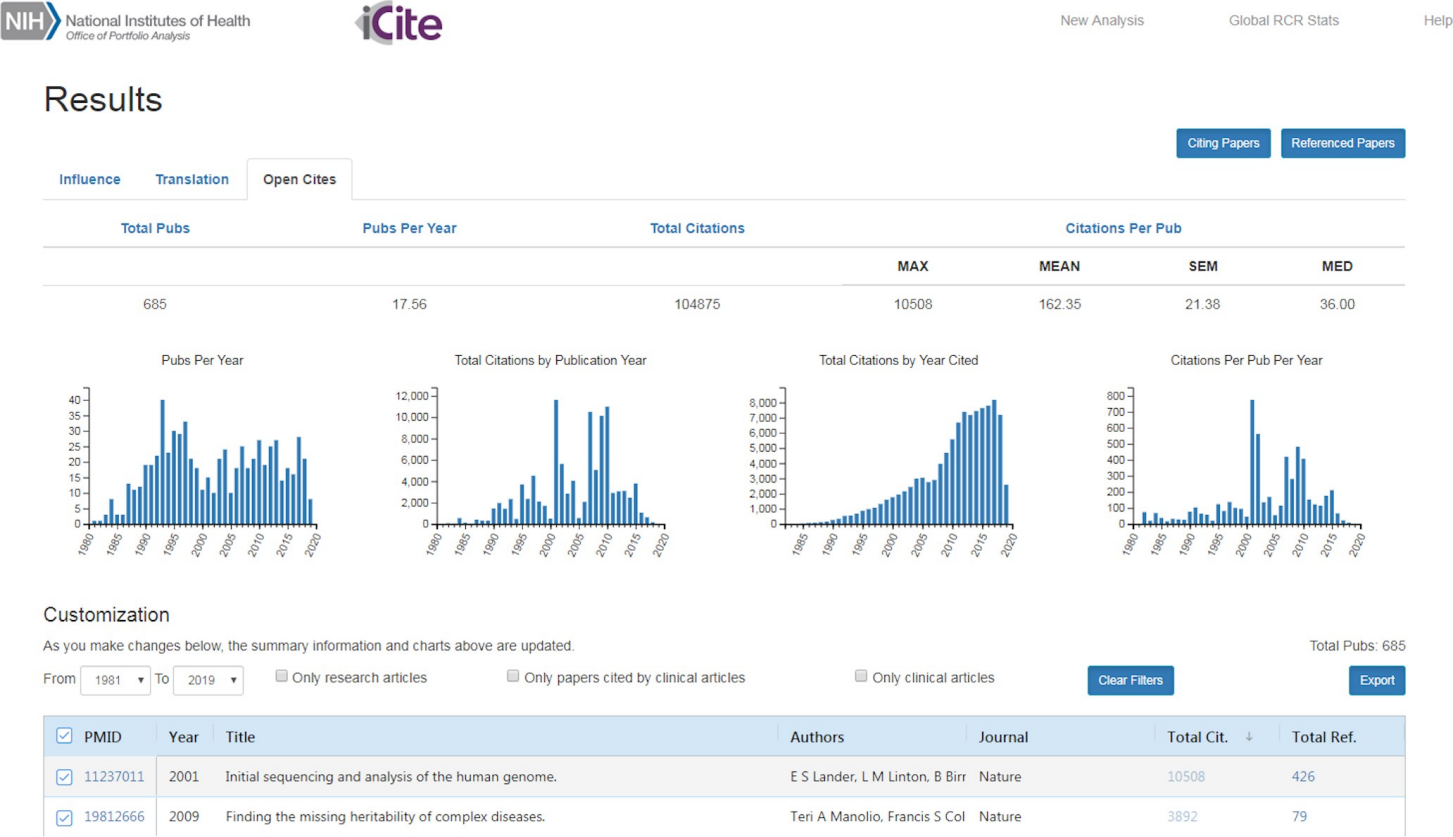
Screen capture of the *iCite* web interface to open citation data. The Open Citations module of *iCite* displays portfolio-level data in a summary table (top) and charts beneath the table. Charts provide visualization of publications over time (left), total citations per year by the publication year of the referenced article (center left), total citations per year by the publication year of the citing article (center right), and average citations per article in each publication year (right). Article-level information is shown on bottom and includes links to the PubMed records of the citing and referenced papers.

At the time of writing (July 2019), the NIH-OCC comprises over 420,000,000 citation links between articles published in PubMed (Fig 1A). The major limitation of the NIH-OCC is that, as part of *iCite*, it has been developed with a biomedical focus; at present, its citation universe is restricted to PubMed-to-PubMed citation links. The largest contribution comes from CrossRef, followed by the NLM, and finally our prototype machine learning pipeline that extracts references from full-text articles (Fig 1B). Although references from the machine learning pipeline represent a small fraction of the total at present, we expect this to increase over time as new papers are identified and processed. Data can be accessed through the *iCite* web interface (https://icite.od.nih.gov/; Fig 2), the *iCite* API (https://icite.od.nih.gov/api), or through bulk downloads (DOI: 10.35092/yhjc.c.4586573).

## Machine learning pipeline for full-text articles

Our data pipeline starts with the identification of full-text articles that have been made freely available on the internet and do not require an institutional library subscription to access. This was first accomplished by identifying journals with open access policies after an embargo period. We also leveraged the recent efforts of Unpaywall [16], which has identified freely available full text at scale, and included these publications in our dataset. Central to our pipeline is our Citation Resolution Service, which accepts unstructured citation text through an API and returns a matched article along with information about which fields from that paper were present (e.g., journal name, author name, title words). The service takes each citation and tokenizes the input to query the search index and find the publications with the highest percentage of matched terms. The scoring algorithm is then run on the candidates to find the best matches by checking fields such as title, authors, and journal name against the input text. Although a general-purpose pipeline is in development, we initially developed a workflow that trained machine learning models on individual journals in order to take advantage of regularities in Portable Document Format (PDF) formatting. For each journal, our workflow was as follows:

1. Harvest PDFs from open sources and convert to structured XML with the open source Cermine tool [24].
2. For papers in the journal that were NIH funded, generate positive training data from text that resolves to previously matched citations in PMC. Combine this with negative training data sampled from other parts of the PDF to train a Long Short-Term Memory (LSTM) recurrent neural network model that discriminates between reference text and other text in the scientific article.
3. Pass LSTM-identified references to the Citation Resolution Service. To filter out any remaining false positives, use PMC data as gold standards to train a Random Forest on the feedback received from the Citation Resolution Service.

This prototype approach yielded excellent precision and recall (both 0.98) in extracting and resolving references when the models were trained on papers from the same journal. Because we used references previously indexed in PMC as gold standards, any false negatives in that dataset would be flagged as false positives in ours; manual inspection of our false positives indicated that over 90% of these were actually false negatives in the gold standards or resolution to a transient duplicate entry of an article (duplicates are typically later identified and removed by PubMed). For papers identified through Unpaywall, which come from a variety of journals, recall across different batches dropped to between 0.78 and 0.89 while precision remained constant. This occurred because more text was filtered by the LSTM, perhaps indicating additional uncertainty about what references look like in a corpus of papers from a variety of journal formats. Whether identified through aggregation services or via permissive journal policies, however, the rapidly increasing number of freely available full-text articles [16] promises to be a rich source of open citation data going forward.

## Future directions

Our prototype machine learning pipeline currently re-trains for each data source. We are building a general-purpose, deep learning reference parser that can both take advantage of recognizable formatting regularities and gracefully handle unknown full-text formats. The comprehensiveness of citation coverage in *iCite* will also benefit from planned future updates that will incorporate reference data from expanded open data sources such as preprint servers and fields of research not indexed in PubMed. We are also engaged in research to predict, in the absence of full text information, which articles are likely to be referenced, based on information present in the local network structure. Finally, we are using the NIH-OCC to develop new artificial intelligence (AI) approaches to generate a high-resolution map of the biomedical research landscape, identify emerging areas, and improve the effectiveness of data-driven decision-making at the NIH. Outside the NIH, open citations may help to power tools and services that do not yet exist, such as next-generation literature recommendation engines. Using the NIH-OCC as the source of citation data means that this work will be transparent and reproducible.

## Abbreviations

AI: artificial intelligence
API: Application Programming Interface
LSTM: Long Short-Term Memory
NIH: National Institutes of Health
NIH-OCC: NIH Open Citation Collection
NIH OPA: NIH Office of Portfolio Analysis
NLM: National Library of Medicine
PDF: Portable Document Format; PMC, PubMed Central

## References

[1] Hawking S. On the shoulders of giants: the great works of physics and astronomy. Philadelphia: Running Press; 2002 xiii, 1264 p. p.

[2] Patterson M, MacCallum C. Inside eLife [Internet]: eLife. 2017 Available from: https://elifesciences.org/inside-elife/45f0e7ed/setting-your-cites-on-open. [cited 2019 July 25].

[3] Patterson M, MacCallum C. PLoS Biologue [Internet]: PLoS. 2017 Available from: https://blogs.plos.org/biologue/2017/04/07/setting-your-cites-on-open/. [cited 2019 July 25].

[4] Fortunato S, Bergstrom CT, Borner K, Evans JA, Helbing D, Milojevic S, et al Science of science. Science. 2018;359(6379). 10.1126/science.aao0185 29496846 PMC5949209

[5] Ioannidis JP, Fanelli D, Dunne DD, Goodman SN. Meta-research: Evaluation and Improvement of Research Methods and Practices. PLoS Biol. 2015;13(10):e1002264 10.1371/journal.pbio.1002264 26431313 PMC4592065

[6] Shotton D. Funders should mandate open citations. Nature. 2018;553(7687):129–. 10.1038/d41586-018-00104-7 32094762

[7] Initiative for Open Citations (I4OC) launches with early success [Internet]. Initiative for Open Citations; 2017 Available from: https://i4oc.org. [cited 2019 June 03].

[8] Spector JM, Harrison RS, Fishman MC. Fundamental science behind today’s important medicines. Science Translational Medicine. 2018;10(438). 10.1126/scitranslmed.aaq1787 29695453

[9] iCite: National Institutes of Health; [cited 2019 Aug 15]. Available from: https://icite.od.nih.gov/.

[10] Hutchins BI, Yuan X, Anderson JM, Santangelo GM. Relative Citation Ratio (RCR): A New Metric That Uses Citation Rates to Measure Influence at the Article Level. PLoS Biol. 2016;14(9):e1002541 10.1371/journal.pbio.1002541 27599104 PMC5012559

[11] Hutchins BI, Hoppe TA, Meseroll RA, Anderson JM, Santangelo GM. Additional support for RCR: A validated article-level measure of scientific influence. PLoS Biol. 2017;15(10):e2003552 10.1371/journal.pbio.2003552 28968381 PMC5624567

[12] PubMed Central National Library of Medicine: National Institutes of Health. Available from: https://www.ncbi.nlm.nih.gov/pmc/. [cited 2019 Aug 15].

[13] MEDLINE^®^: Description of the Database. Available from: https://www.nlm.nih.gov/bsd/medline.html. [cited 2019 Aug 15].

[14] Entrez Programming Utilities Help. National Center for Biotechnology Information (US): National Library of Medicine; 2010. 10.3163/1536-5050.98.2.012 PMC2859267 20428285

[15] CrossRef. Available from: https://www.crossref.org/. [cited 0219 Aug 15].

[16] Piwowar H, Priem J, Lariviere V, Alperin JP, Matthias L, Norlander B, et al The state of OA: a large-scale analysis of the prevalence and impact of Open Access articles. PeerJ. 2018;6:e4375 10.7717/peerj.4375 29456894 PMC5815332

[17] Open citations: A letter from the scientometric community to scholarly publishers [Internet]. International Society for Informetrics and Scientometrics; 2017 Available from: http://issi-society.org/open-citations-letter. [cited 2019 June 03].

[18] Singh Chawla D. Open-access row prompts editorial board of Elsevier journal to resign. Nature. 2019 10.1038/d41586-019-00135-8

[19] Sinatra R, Wang D, Deville P, Song C, Barabasi AL. Quantifying the evolution of individual scientific impact. Science. 2016;354(6312). 10.1126/science.aaf5239 . 27811240

[20] Wang D, Song C, Barabasi AL. Quantifying long-term scientific impact. Science. 2013;342(6154):127–32. 10.1126/science.1237825 . 24092745

[21] Wu L, Wang D, Evans JA. Large teams develop and small teams disrupt science and technology. Nature. 2019;566(7744):378–82. 10.1038/s41586-019-0941-9 . 30760923

[22] Stoeger T, Gerlach M, Morimoto RI, Nunes Amaral LA. Large-scale investigation of the reasons why potentially important genes are ignored. PLoS Biol. 2018;16(9):e2006643 10.1371/journal.pbio.2006643 30226837 PMC6143198

[23] Hutchins BI, Davis MT, Meseroll RA, Santangelo GM. Predicting translational progress in biomedical research. PLoS Biol. 2019;17(10):e3000416 10.1371/journal.pbio.3000416 PMC6786525 31600189

[24] Tkaczyk D, Szostek P, Fedoryszak M, Dendek PJ, Bolikowski L. CERMINE: automatic extraction of structured metadata from scientific literature. Int J Doc Anal Recog. 2015;18(4):317–35. 10.1007/s10032-015-0249-8 WOS:000368507400003.

